# Dr. Anthony Harris on his comedy, career, and the mentorship North Star

**DOI:** 10.1017/ash.2026.10407

**Published:** 2026-05-28

**Authors:** Anthony D. Harris

**Affiliations:** Department of Epidemiology and Public Health, University of Maryland School of Medicine, Baltimore, MD, USA

## Abstract

Dr. Anthony Harris is Professor of Epidemiology and Public Health and Internal Medicine, and Head of the Division Genomic Epidemiology and Clinical Outcomes of Health Care Outcomes Research. He is an infectious disease physician and epidemiologist whose research interests include emerging pathogens, antimicrobial-resistant bacteria, hospital epidemiology/infection control, epidemiologic methods in infectious diseases, and medical informatics. He has published over 360 papers. He has current or has had funding from the NIH, CDC, VA, and AHRQ to study antibiotic resistance and hospital epidemiology. He is extremely proud of his mentoring track record.

## You are very funny and have respectable skills in stand-up comedy. How has comedy played a role in your career as a physician, epidemiologist, scientist, and mentor?

Thanks for the compliment—I think my family would not agree.

It really arose from trying to be the best educator I could. Early on, I was tasked with teaching epidemiology and statistics to medical students—and as you can imagine, that’s not an area they immediately think is important. So, as an educator, you have to figure out the best way to relay those messages. I started using comedy in lectures to engage them and, hopefully, help them actually learn epidemiology and statistics.

Whether it’s comedy or something else, especially in this era of changing medical curricula, becoming a more effective communicator is key. I keep trying to hone my comedy skills to improve my overall communication and teaching.

## When you received the invitation for the 2025 SHEA lectureship, how did you come to select mentorship as your North Star?

When you receive this kind of award, you’re humbled. You look at the people who’ve come before you—truly great people—and you feel this enormous pressure to deliver a good lecture and to live up to the legends who have received this award.

I also felt pressure related to how difficult things have been for our field. Let’s be honest—there’s a lot of scientific denialism, and it directly affects infectious diseases. Infectious diseases is in a tough situation right now and I did not have answers about how to get out of this mess.

So, I asked myself: how can I make this talk meaningful? Many prior talks in this meeting have focused on the “doomsday” aspects of where things are currently and may be headed. I wondered if there was a way to do something a bit more uplifting.

Mentorship has brought me a tremendous amount of joy—and hopefully to some of my mentees as well. So, I thought focusing on mentorship could bring some positivity to the meeting while still being meaningful.

## One of your main takeaways from medical school and residency was the importance of thoroughness and going deep. How has this shaped your approach as a physician and researcher?

This message is really important—and honestly, I wish I followed it myself more consistently day to day. But the times I do go deep, I find it incredibly gratifying, and the end product is better.

For example, for clinicians: when you have a difficult case, it’s worth shifting your mindset, putting other things aside, and saying, “*This is a case where I need to go down to radiology and review the MRI,* or *I need to sit down with the primary team and really talk this through or I need to reach out to a colleague within my institution or even outside of my institution.”* When I spend that extra 30–45 minutes, I rarely regret it. I often uncover something—whether from the radiologist or the team—that improves patient care. That’s incredibly satisfying.

The same going-deep mindset applies to research. When I commit to being more thorough—whether in writing or double-checking analyses, I often uncover insights that improve the science.

I think one of our biggest challenges as a field is time and time management. We simply don’t have enough of it to do things as thoroughly as we’d like. And I suspect we’d enjoy our work more if we could create that time to do things more thoroughly.

## We were intrigued by an anecdote in your presentation about an early mentor, Dr. Tom Hutchinson, who reinvented himself every decade. If you could reinvent yourself in 10 years, what would you become?

One thing I learned from Tom is that he didn’t care what others thought about his career trajectory—he focused on what gave him purpose. I’m not sure I’m as strong in that regard but aspire to be.

I do think about reinvention a lot lately. Ideally, I’d love to see a resurgence in research and play a role in helping others do meaningful work—work that truly changes things.

For example, I’d love to contribute, even in a small way, to people tackling climate change—despite it being outside infectious diseases.

I also think about periods where I might reinvent myself clinically—really focusing deeply on one area and becoming better in that clinical space. Those are a couple of reinvention directions I’ve considered.

## Themes in your talk included “if you don’t shoot, you don’t score,” collaboration, and connection. How have these sustained you during difficult times?

Let me start with connection and appreciation—in my opinion, that’s the most important response to your question. We all work hard—40, 50, 60 hours a week. If you’re committing that much time at work, it must be more than transactional. You want relationships at work that are meaningful—maybe not at the level of family, but close.

You want to come to work excited to see people, to feel connected, to feel appreciated—and to appreciate others. That makes the difficult times much more manageable and meaningful.

On the collaboration theme—I’ve been incredibly lucky. I’ve worked with people who think completely differently from me, bring a different skillset, and that’s been invaluable.

One example: through an NIH K24 mentoring grant, I met Sean Barnes, an aerospace engineer who was obtaining a PhD in computational math. I sat on his committee thinking, *I am completely out of my depth.* But over time, we learned from each other. That relationship eventually led to him working at Netflix—and suddenly, I found myself consulting for Netflix.

Another example: an orthopedic trauma surgeon introduced me to surgical site infection rates among trauma patients that I didn’t fully appreciate. In my defense, the reason I did not recognize them is that these were not procedures that infection control and pay-for-performance traditionally focused on. This collaboration led to gratifying randomized trials in orthopedic trauma—outside traditional ID work.

These kinds of “bizarro” collaborations—where everyone is a bit out of their depth—have been incredibly rewarding.

## How would you characterize your mentorship style?

Day-to-day mentorship is about time—and there’s never enough of it.

Mentees often have significant needs, and mentors are stretched thin. So, it’s critical to empower mentees to articulate what they need—and then make the time to support their goals.

In terms of what I treasure most: long-term relationships between mentors and mentees.

I feel incredibly lucky to have mentees with whom I’ve maintained relationships long after the formal mentorship period ended—people like Sean Barnes, JJ Furuno, Jessina McGregor, and Max Masnick.

And I hope to build similar long-term relationships with current mentees like Jon Baghdadi, Katie Goodman, and Lyndsay O’Hara.

## What areas are you working to improve as a mentor?

Mentors must keep evolving. Problems arise when we think too highly of ourselves and stop self-reflecting.

It’s also hard because mentees may not feel comfortable pointing out mentors’ weaknesses.

For me, listening is a big area I am trying to improve. Phone distraction is a real problem these days. When you’re overextended, your mind is racing instead of being present with your mentee.

Another challenge is recognizing when a mentorship relationship isn’t working—and having the clarity to step back instead of over-analyzing it. And try not to take it personally that a mentor-mentee relationship is not working.

## You conclude with a call to action around advancing the science of healthcare epidemiology. Can you describe your “roadmap” toward achieving this goal?

That part of the talk could’ve been an entire lecture on its own, so I apologize for it being such a brief overview.

One area I advocated for expanding is emulation trials, which are essentially large database studies. With the explosion of data, there are many opportunities to do meaningful research without massive cost. Platforms like Premier, Vizient, and Cosmos offer these opportunities.

Another area I propose expanding into is pragmatic trials. Pragmatic trials are more complex but represent the future, embedding randomized trials into electronic health records to answer real-world clinical questions. That will require infrastructure, IRB coordination, and technical expertise—but it’s where we’re headed.

## What did the IDWeek 2025 SHEA lectureship mean to your family?

It meant a lot. My wife and three kids were very proud—they know how much SHEA means to me and how much SHEA has helped me.

When I showed them how I incorporated them into the talk, it became a unifying moment.

My eldest daughter said, “Wow, you’re a great speaker.” I told her in a hopefully self-deprecating tone, “I’ve been doing this for 30 years.”

It was a nice moment—mutual pride on both sides.

## What books are on your nightstand?

One is *Putting Happiness to Work* by Eric Karpinski, building on Shawn Achor’s work out of Harvard. It explores how to create happier work environments—through appreciation, connection, and purpose.

Another is Getting to Yes—a classic on negotiation and conflict resolution, which I think ID physicians often underappreciate.

And Good to Great—a more data-driven look at how organizations improve performance.

## What do you do to unwind?

I unwind by exercising. I like skiing and playing golf. But I also like training for sprint duathlons and sprint triathlons and thus bike, run and swim.

I like watching comedies. But I realized that there was a 10–15-year stretch where I didn’t watch movies or TV as I was raising kids. So now I’m catching up on some gems—about 20 years late. I’ve really enjoyed Scrubs and Arrested Development, both new to me.

And I’m from Canada so I also watch a lot of ice hockey.

## Who are your top comedians?

I like Dave Chappelle—he’s controversial, but he’s incredibly funny.

Also Gary Gulman—he has a lot of depth to his comedy.

And finally, Zarna Garg, who I’ve seen live a couple of times, she’s fantastic. She’s really, funny.

